# Security Analysis of Machine Learning-Based PUF Enrollment Protocols: A Review

**DOI:** 10.3390/s21248415

**Published:** 2021-12-16

**Authors:** Sameh Khalfaoui, Jean Leneutre, Arthur Villard, Ivan Gazeau, Jingxuan Ma, Pascal Urien

**Affiliations:** 1 EDF R&D, 7 Boulevard Gaspard Monge, 91120 Palaiseau, France; arthur.villard@edf.fr (A.V.); ivan.gazeau@edf.fr (I.G.); jingxuan.ma@edf.fr (J.M.); 2 LTCI, Télécom Paris, Institut Polytechnique de Paris, 19 Place Marguerite Perey, 91120 Palaiseau, France; jean.leneutre@telecom-paris.fr (J.L.); pascal.urien@telecom-paris.fr (P.U.)

**Keywords:** Internet of Things, authentication, physical unclonable function, machine learning

## Abstract

The demand for Internet of Things services is increasing exponentially, and consequently a large number of devices are being deployed. To efficiently authenticate these objects, the use of physical unclonable functions (PUFs) has been introduced as a promising solution for the resource-constrained nature of these devices. The use of machine learning PUF models has been recently proposed to authenticate the IoT objects while reducing the storage space requirement for each device. Nonetheless, the use of a mathematically clonable PUFs requires careful design of the enrollment process. Furthermore, the secrecy of the machine learning models used for PUFs and the scenario of leakage of sensitive information to an adversary due to an insider threat within the organization have not been discussed. In this paper, we review the state-of-the-art model-based PUF enrollment protocols. We identity two architectures of enrollment protocols based on the participating entities and the building blocks that are relevant to the security of the authentication procedure. In addition, we discuss their respective weaknesses with respect to insider and outsider threats. Our work serves as a comprehensive overview of the ML PUF-based methods and provides design guidelines for future enrollment protocol designers.

## 1. Introduction

The deployment of smart sensors is exponentially increasing to cover consumer oriented services and the requirements of industrial scenarios [1]. The high popularity of Internet of Things (IoT) products is pressuring manufacturers to opt for rush to market behavior in order to comply with their clients’ needs. Thus, they tend to overlook the importance of ensuring the security of these resource-constrained devices, which might create a potential attack vector once they are deployed. Moreover, the National Institute of Standards and Technology (NIST) has recently introduced new regulations, NISTIR 8259A [2], for the United States IoT market regarding the security of new devices and the data collection. On the other hand, the European Telecommunications Standards Institute (ETSI) has released similar cybersecurity guidelines, in the ETSI EN 303,645 report [3], for the IoT consumer market in Europe and in the United Kingdom. Manufacturers need more comprehensive and easy-to-adopt security solutions in order to keep pace with the regulations. Therefore, the application of an entity authentication procedure that is suitable for the IoT context is crucial. By doing so, we eliminate the risk related to allowing a malicious object into the network of a user. This secure enrollment process ensures that the communicating IoT nodes are trustworthy.

Numerous entity authentication solutions have been proposed to verify the identity and the origin of the IoT object in question. The identity-based [4] and certificate-based techniques [5] represent promising candidates when combined with a lightweight cryptographic algorithm that is supported by a resource-constrained device. However, we encourage the avoidance of exploiting any pre-established security knowledge between the verifier and the prover to facilitate the integration of our solution with a secure device pairing scheme [6,7]. The no prior secrets condition is motivated by the unfeasibility of managing the scalability issues of public key infrastructure due to the growing numbers of heterogeneous IoT devices. A third possible alternative is to exploit a One-Time Password (OTP) system [8] that authenticates the object using a trusted third party. Nevertheless, this technique requires the IoT device to communicate independently with a remote OTP server. As a consequence, we would prefer to perform the authentication process prior to associating the IoT object with the network of the user. Therefore, the OTP solution would not be compliant with our requirements. A final alternative is to use of a hardware-based enrollment protocol that relies on a secure element such as a PUF [9] onboard the object. This method provides a lightweight and a cost-effective authentication system that is adequate with the IoT context. Several integrated circuit vendors have opted for a hardware-level technology approach for securing the use of the IoT object through physical unclonable functions. These secure hardware elements serve multiple objectives, such as device identification, secure key management and secure boot functionality. This technology has been applied to IoT products, but it can also play a major role in the security systems used in other industrial areas, such as the vehicular context, as discussed in [9]. This role can cover, for example, the vehicle component identification [10] or cryptographic key management for securing a vehicular ad hoc network [9].

Unfortunately, a growing number of the recently proposed PUFs, such as the Interpose-PUF [11] and the Double Arbiter PUF (DAPUF) [12], have been proven vulnerable against a variety of machine learning (ML) attacks that aim at modeling their behavior by collecting a sufficient number of challenge–response pairs (CRP) [13,14]. Therefore, several enrollment protocols have intentionally exploited some vulnerable PUF architectures to create ML models that simulate their behavior [15,16]. The work of Pour et al. [17] briefly discusses the benefits of exploiting these modeling methods in an industrial scenario. These advantages include reducing the time that is required to enroll a large number of devices and the storage space that should be used to store the challenge–response pairs. As a consequence, a server can efficiently many IoT devices. The existing reviews of PUF-based enrollment procedures tend to focus on the traditional use of these hardware circuits through the storage of the CRPs [18,19]. Other reviews concentrate on reviewing the vulnerabilities of these PUF architectures to ML modeling attacks [20,21,22]. However, we have noticed that they overlook the exploitation of these ML modeling techniques in order to reduce the required storage space while maintaining the same level of security.

In this paper, we provide an in depth overview of the state-of-the-art model-based PUF enrollment protocols. We classify the existing proposals based on two architectures. In addition, we describe the different components of the protocols and we discuss their respective weaknesses. Additionally, we evaluate the robustness of the identified enrollment protocols against an insider threat scenario that targets the secrecy of the given PUF ML model. Our paper serves as a comprehensive overview of the scalable PUF-based methods that have been used so far to perform the enrollment procedures of IoT objects.

The rest of the paper is organized as follows. Section 2 introduces the basics of the PUF technology and the different machine learning techniques that are applied to model these circuits. Section 3 presents the two enrollment architectures and details their building blocks. Section 4 provides an extensive study of the existing model-based PUF enrollment protocols in light of the proposed architectures. Section 5 discusses the advantages and the limitations of these architectures, alongside of the weaknesses identified in the described schemes. Furthermore, it outlines the impacts of the insider attack scenario on the security of the authentication process and provides future research directions to mitigate the threats. Lastly, Section 6 concludes the work.

## 2. Preliminaries

In this section, we provide background on the physical unclonable functions, and we describe their most commonly used constructions for authenticating physical systems. Furthermore, we introduce a selection of the most effective machine learning techniques for modeling the behavior of PUF circuits.

### 2.1. Physical Unclonable Function

A physical unclonable function is a secure element that identifies, in a unique manner, a specific device through a challenge–response process. This paired information represents the pattern of responses when we have a set of specific challenges as inputs. This function has to be unclonable and unique for each device, since it relies on physical randomness that can be either explicitly introduced or intrinsically present in the physical system [23]. The micro-variations in the hardware system allow the same construction of a PUF to provide unique responses when deployed on different circuits. Thus, these variations play the role of the seed in a random response generator.

There are two major categories of PUFs based on the source of the randomness. The first category of PUFs, referred to as *electronic PUFs*, rely on a number of micro-physical parameters that are hidden from the physical observation inside the electronic circuit. These parameters can be detected only when they are needed to produce the unique responses. These variables include the time, the frequency, the current or the voltage, the bistable states and the capacitance [24]. The second category of PUFs, referred to as *non-electronic PUFs*, includes the PUF elements that rely on unique characteristics of the physical system in a non-electronic manner, such as the use of light in optical PUFs [25] and the radio variations in the RF-PUF [26]. Readers that are eager to learn more about the different PUF architectures can consult the review in [27].

The electronic PUF elements can be further classified into two categories: *strong PUFs* and *weak PUFs*. Strong PUFs provide many challenge–response pairs, which makes them suitable for the authentication operations. This is explained by the possibility of conducting numerous authentication attempts using different CRPs in each session without the need to reuse the same credentials. Thus, they represent interesting candidate solutions in the context of multi-user IoT objects. Weak PUFs provide fewer CRPs. However, these PUFs have been increasingly popular as internal key generators [28,29]. In this work, we focus on the authentication protocols that are based on strong PUFs.

#### 2.1.1. Arbiter PUF

**Figure 1:**
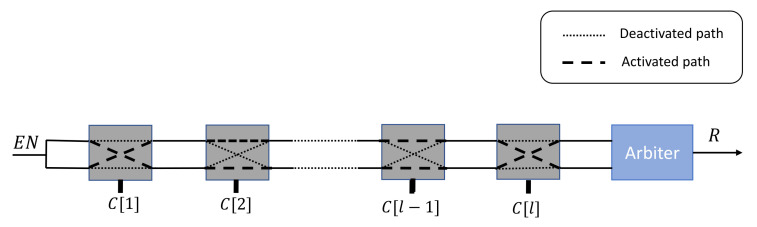
Arbiter PUF architecture.

The Arbiter PUF [30] is one of the most popular electronic PUFs that are exploited for authentication operations. This PUF’s architecture is based on a comparison of the travel times of two electrical signals propagating down two symmetrical paths. The uniqueness of the responses is based on the manufacturing variations in the creation of these two paths. This PUF is constructed using a pre-determined number of 22 cells that connect these paths. The choice of connection routes depends entirely on the *l* challenge bits C[x],x∈[1,l]. Finally, the arbiter component decides which signals has arrived first, and accordingly outputs the associated binary response, as illustrated in Figure 1.

#### 2.1.2. XOR Arbiter PUF

**Figure 2:**
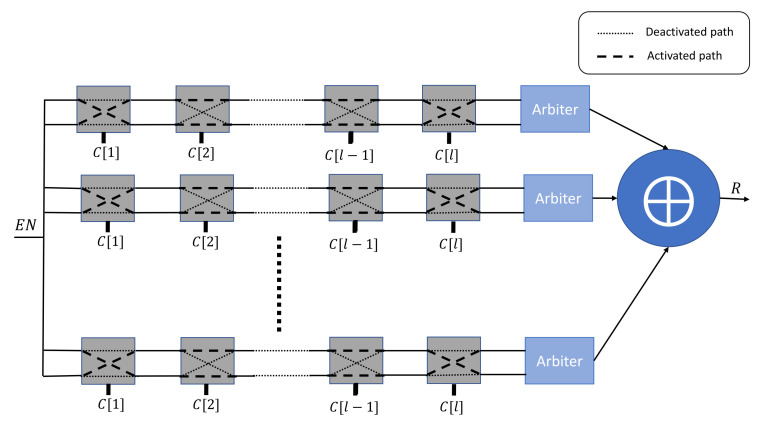
*n*-XOR Arbiter PUF architecture.

This PUF architecture is a variant of the previously described Arbiter PUF. It has been developed as way to enhance the complexity of the mapping function between the input challenges and the output responses. As highlighted in Figure 2, this construction uses *n* independent Arbiter PUFs, and it applies an XOR operation on their individual responses to obtain the output response *R*. However, the stability of the responses is highly affected by the number *n* of applied Arbiter PUFs.

**Figure 3:**
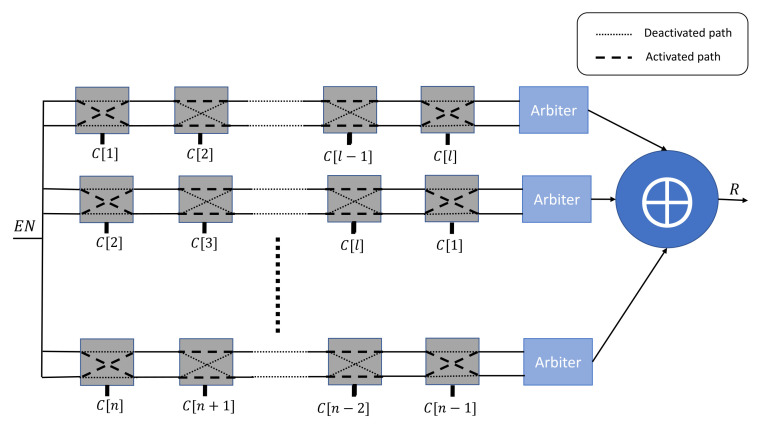
*n*-XOR Arbiter PUF variant with a derivative challenge for each stage.

Yu et al. [31] has presented another variant of the XOR Arbiter PUF by applying a different challenge at each stage. *n* challenges can be constructed by applying a linear-feedback shift register (LFSR) to the received root challenge *C*, as shown in Figure 3.

#### 2.1.3. Logically Reconfigurable PUF

The Logically Reconfigurable PUF (LR-PUF) [32] represents a secure hardware element that has the ability to change its challenge–response behavior. The reconfigurability aspects can be achieved in the context of integrated circuits through the use of a field-programmable gate array (FPGA). These PUF circuits should guarantee two properties: *forward* and *backward-unpredictability*. The former property assures that the challenge–response pairs collected before the reconfiguration is invalid. Thus, the adversary cannot model the current PUF behavior through the use of previously collected CRPs. The latter property guarantees that an adversary with access to the current reconfigured PUF cannot estimates the responses before the reconfiguration. The work of Liu et al. [33] has identified two type of LR-PUFs: *circuit-based reconfigurable PUFs (C-RPUFs)* and *algorithm-based reconfigurable PUFs (A-RPUFs)*. The former category uses reconfigurable components onboard the circuit to change the original construction. Thus, this hardware level modification changes the behavior of the PUF. The latter category keeps the original hardware components, and instead, it applies a configurable algorithm to change the mapping between the challenges and the responses.

### 2.2. Modeling of PUF Designs

#### 2.2.1. Logistic Regression

Logistic regression (LR) is a well-known supervised learning technique. This method models the probability of a discrete outcome that is associated with a specific input variable. The LR learning algorithm is based on the sigmoid function and a set of weights that are learned by using the training dataset. The logistic regression technique is commonly used for the binary classification problems. Therefore, this methodology has been applied, in [14,21,22], to model the behavior of a binary output PUF such as the Arbiter PUF variants, described in Section 2.1.1 and Section 2.1.2.

The resilient propagation (RProp) [34] has been an increasingly popular algorithm to optimize the weight coefficients of the logistic regression technique. This is due to its ability to dynamically adapt the step size, independently, for each weight. This technique has been applied in the work of Rührmair et al. [13,35] to model the *x*-XOR Arbiter PUF with x≤5 and with an accuracy of up to 98%. Furthermore, the work of Khalafalla and Gebotys [22] has exploited a LR learning technique with a linear decision boundary against a more complex Arbiter PUF variant (DAPUF [12]). This method has yielded an enhanced modeling accuracy of up to 99% with less challenge–response pairs and with cheaper computing resources.

#### 2.2.2. Support Vector Machine

**Figure 4:**
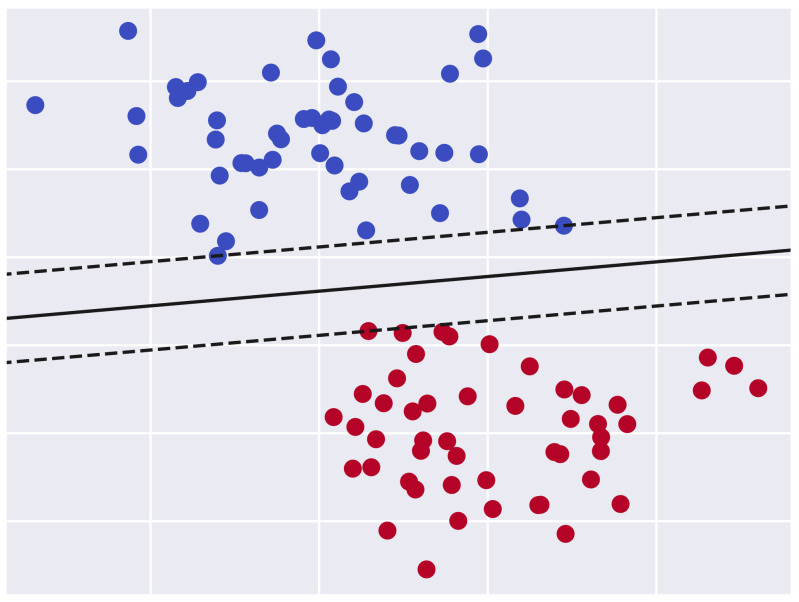
Binary classification problem using the support vector machine algorithm.

The support vector machine (SVM) algorithm [36] has been widely used in classification tasks. The objective of this technique is to find an optimal hyperplane in a N-dimentional space that separates the data points. This hyperplane should classify the data points in a way that maximizes the distance between the identified classes. The Figure 4 illustrates a binary classification problem where the optimal hyperplane is represented as a continuous line. However, the dashed lines represent the other candidate hyperplanes that do not provide the maximum margin between the two classes. Due to the popularity of the SVM algorithm in the binary classification tasks, it has been used in numerous studies [13,22,35,37,38] to model some variants of the Arbiter PUF with limited complexity.

#### 2.2.3. Artificial Neural Networks

**Figure 5:**
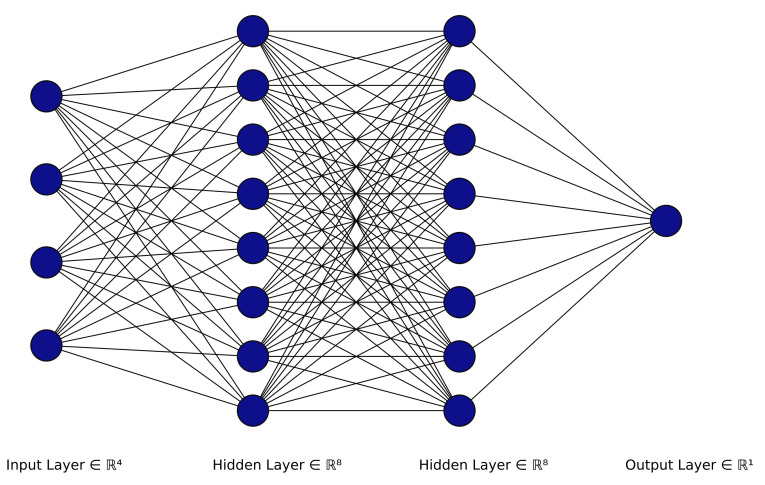
Artificial neural network architecture.

The artificial neural network (ANN) [39] is a system that imitates the function of the human brain through the use of multiple artificial neurons. This system consists of a number of neuron layers that are referred to as an input layer, one or multiple hidden layers and the classifier layer, as illustrated in Figure 5. Each neuron in the network is connected to another and has an associated weight and a threshold. These parameters are updated over time based on the training data to improve the prediction accuracy of the neural network model.

The ANNs that consist of single hidden layers are referred to as single layer perceptrons (SLPs) and they are only applicable in the case of linearly separable data. Therefore, multiple layer perceptrons (MLPs) are used in the case of non-linear problems. In the context of PUF modeling, a great body of work exploits the power of these model to either attack the state-of-the-art PUF constructions or to demonstrate their resiliency against ML modeling attempts. Unfortunately, a growing number of the proposed ML-resistant PUFs, such as the Interpose-PUF [11] and the 9-Xor Arbiter PUF [40], have been proven vulnerable against a variety of ANN attacks that aim at modeling their behavior by accessing a sufficient number of challenge–response pairs [14,41,42,43].

#### 2.2.4. Evolutionary Strategies

The evolutionary strategies (ES) are stochastic techniques for the numerical optimization of non-linear and non-convex learning problems. This class of ML methodologies is inspired by the biological evolution of individuals due to specific environmental conditions, also referred to as the survival of the fittest. In the context of the PUF technology, this individual is represented by a vector of runtime delays in the circuit components. The algorithm generates random PUF instances that are referred to as parents. They are tested to check the resemblance with the target PUF responses using the fitness function that should be specified by the user. Afterwards, the child instances inherent the parents’ characteristics (delay vectors in the case of Arbiter PUFs) with minor random modifications, and the resemblance process is conducted for many generations.

**Figure 6:**
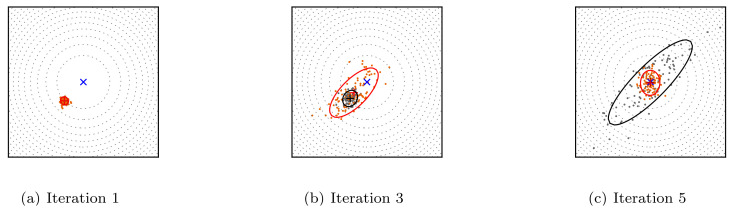
The optimizationproblem of a two-dimensional linear function using the CMA-ES algorithm. The orange and gray dots represent the distributions of the child and the parent populations.

The Covariance Matrix Adaptation Evolutionary Strategy (CMA-ES) [44] is one of the most known ES that performs well on complex optimization problems. This variant uses the covariance matrix to adjust the dependencies between the variables in the normal distribution. Figure 6 illustrates the steps of the CMA-ES technique. The algorithm starts by generating random parent individuals according to the normal distribution. Afterwards, the fittest candidates are selected based on a specific fitness function and the algorithm updates its internal parameters. Finally, a new population is generated based on the previous updates, and the process is repeated until convergence.

The CMA-ES technique has been widely used to model complex PUF architectures such as Interpose-PUF [11]. This algorithm was also applied in the work of Becker [45] to attack the two versions of the Slender-PUF [15,46]. The adversary targeted the response obfuscation mechanism in order to use the obfuscated CRPs to efficiently model PUF circuit.

#### 2.2.5. Other Machine Learning Techniques

Other ML techniques such as decision tree, random forest and naïve Bayes classification have been applied to model the behavior of the PUF circuits [21,47]. These techniques have been adopted to address the signal classification and networking problems [48,49]. However, in the work of Kroger et al. [47], they were demonstrated to be less effective in comparison to the previously described algorithms when using a relatively large dataset of challenge–response pairs (more than 400 CRPs). On the other hand, these techniques achieved a better accuracy wh training on a small dataset (less than 400 CRPs).

## 3. Model-Based PUF Authentication Procedure

In this section, we describe the authentication process of an IoT object based on the use of a mathematically clonable PUF based on a number of ML techniques. The procedure consists of multiple entities that participate in verifying the identity of this particular device. These entities constitute two generic architectures that represent the steps of an enrollment protocol. Each of these components are defined and characterized based on the roles and the modules that are specified by the protocol designer. The building block diagrams in these two architectures can help to design and assess independently the system components of these schemes with respect to the adopted threat model. Furthermore, we provide global insights into the enrollment process and the components. This section introduces the insider threat model in the enrollment process, which is usually overlooked by designers. This model aims at assessing the robustness of the protocols against the scenario of leakage of a secret PUF model to an adversary.

### 3.1. Enrollment Architectures

The existing model-based PUF authentication protocols can be classified based on two generic architectures that we refer to them as three-component (3CE) and four-component (4CE) enrollment procedures. As the name states, the former approach requires the existence of three main high-level roles:

- **Prover:** The IoT object that needs to be enrolled in the network of the user based on the PUF hardware onboard it.
- **Communication channel (CC):** The communication channel for the components.
- **Authentication server (AS):** The entity that manages the storage and the accessibility to the PUF model. Furthermore, it performs the enrollment procedure with the prover as the root of trust (RoT) [50] in the authentication process through the chosen communication channel.

**Figure 7:**
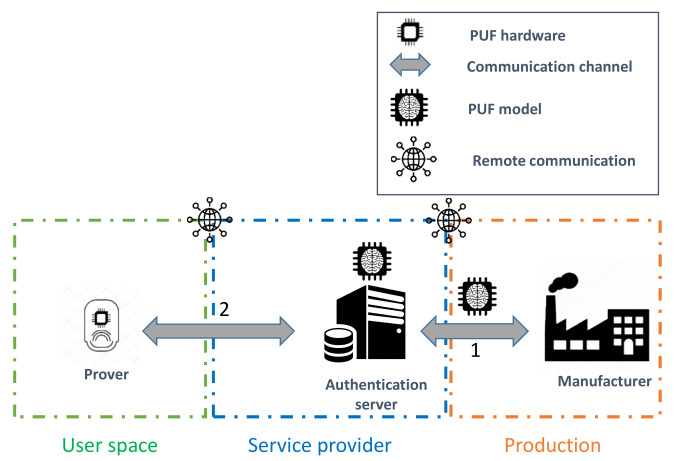
Three-component enrollment procedure.

This approach typically requires the unauthenticated IoT object to connect to the network of the user to remotely communicate with the authentication server, as illustrated in Figure 7. On the other hand, the latter architecture is slightly different, since it exploits a delegated root of trust (RoT) [50] role, referred to as the verifier. The four components of this approach are described as follows:

- **Prover:** The IoT object that needs to be enrolled in the network of the user based on the PUF hardware onboard it.
- **Communication channel (CC):** The communication channel for the components.
- **Verifier:** The designated entity that performs the enrollment procedure with the prover on behalf of the RoT in the authentication process through the chosen communication channel. This role and the the authentication server constitute the chain of trust in the enrollment procedure.
- **Authentication server (AS):** The entity that manages the storage and the accessibility to the PUF model. Moreover, it adds the enrolled prover to the list of authorized devices to join the network based on the validation of the verifier.

**Figure 8:**
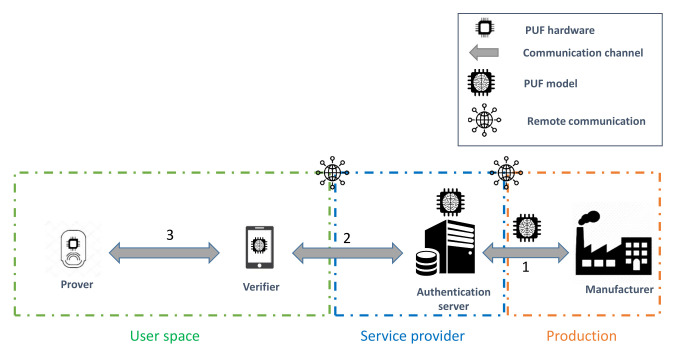
Four-component enrollment procedure.

The delegated root of trust acts as the local challenger of the IoT device, as shown in Figure 8. Therefore, it prevents the risks related to connecting an unauthenticated object to a poorly isolated network. Furthermore, it helps to decrease the communication and computational costs on the server side. Thus, the verifier role enhances the scalability of the enrollment procedure.

### 3.2. Overview of Components

In this subsection, we describe the roles and the modules that constitute each component. These generic elementary units serve as building blocks to the previously introduced architectures.

#### 3.2.1. Prover

The prover role represents the IoT object that holds the PUF hardware. This secure element represents a means to perform the entity authentication procedure. Depending on the adopted enrollment architecture, the IoT device can be given access to the network of the user prior to the authentication process to communicate with the AS, as illustrated in Figure 7. However, in the case of the 4CE approach, the prover is limited to using local communications with the verifier until the successful execution of the enrollment protocol.

**Figure 9:**
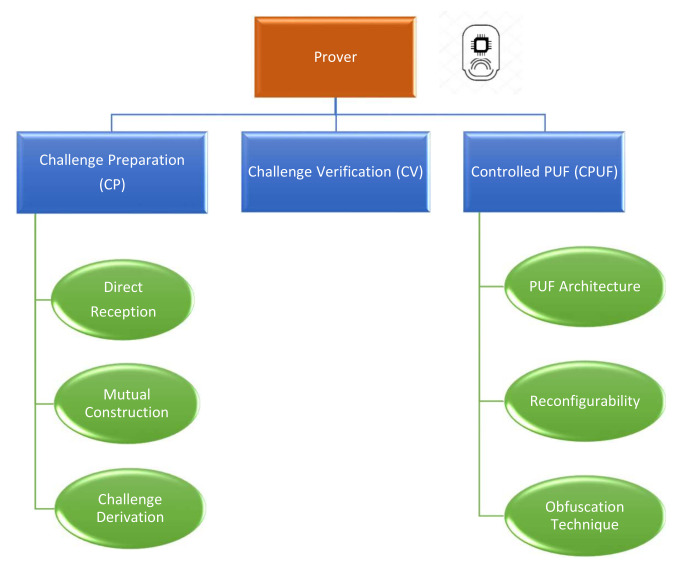
Key elements of the prover role.

The application of a PUF ML model in the protocol design is an admission that this secure element can be mathematically cloned when the adversary has a sufficient number of challenge–response pairs. Therefore, additional protection techniques should be implemented to prevent the attacker from constructing their own precise PUF model. Following the specifications in the work of Maes [51], the added security measures classify this PUF construction as a *Controlled PUF*. The prover role is established based on three main elementary units, as highlighted in Figure 9, that manage the input–output transformation. The three sub-components are as follows:

- **Challenge preparation (CP):** The CP unit is responsible for receiving and preparing the received challenge from the verifier. This part can be split into three main categories:
  − *Direct reception:* The received challenges can be fed directly to the PUF hardware.
  − *Mutual construction:* The prover and the verifier collaborate to compute a common seed to generate the set of challenges. One simple example of this operation is to exchange nonces that are concatenated to find the shared seed value.
  − *Challenge derivation:* The prover receives a single *l*-bit challenge that is manipulated to extract in total a set of *l* challenges. As an example of this operation, the receiver can apply a linear-feedback shift register to the received root challenge.
- **Challenge verification (CV):** The CV unit is responsible for verifying the validity of the challenges that are fed to the PUF hardware. For example, the verification process may aim at ensuring that the received challenges have not been executed before. This technique is considered a means of mitigation against the reliability attack that was proposed in the work of Becker [45].
- **Controlled PUF (CPUF):** The CPUF unit constitutes the most important component on the prover side. This part is responsible for generating and obfuscating the PUF responses. The CPUF has three main aspects:
  i. *PUF architecture:* The chosen PUF construction to be implemented in the prover.
  ii. *Reconfigurability:* This aspect is only discussed in the case of FPGA. The integrated circuit onboard the prover can be reconfigured by the verifier to impose a specific behavior of the PUF.
  iii. *Obfuscation technique:* The specification of the chosen approach to hide the responses from the adversary to prevent any modeling attacks based on the collected CRPs.

#### 3.2.2. Verifier

**Figure 10:**
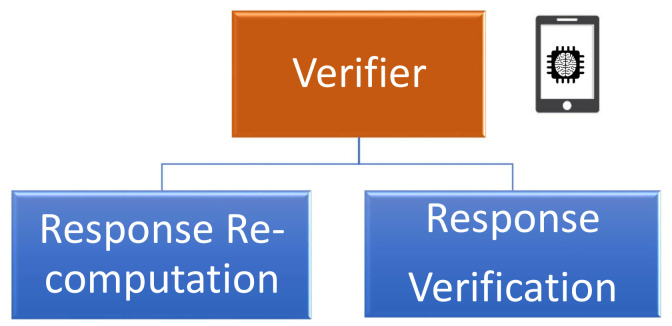
Key elements of the verifier role.

The verifier role is considered as the local root of trust that initiates the challenge–response process with the prover, as illustrated in Figure 8. This component plays a crucial role in generating the enrollment challenges and in verifying the validity of the received obfuscated responses. In this context, the verifier takes advantage of the received PUF model from the authentication server to perform the enrollment process, as illustrated in Figure 10. The verification responsibility can be divided into two main parts:

- **Response re-computation:** The verifier applies the chosen challenges to the PUF model to extract a set of probably approximately correct responses.
- **Response verification:** This process uses the received responses from the prover and the re-computed values from the PUF model to validate the identity of the sender.

#### 3.2.3. Authentication Server

**Figure 11:**
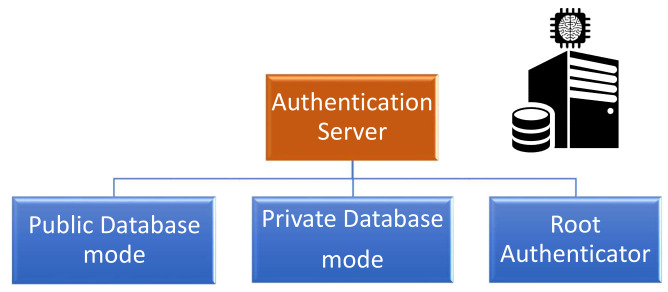
Key elements of the authentication server’s role.

The authentication server is considered as the primary root of trust in both architectures. This component guarantees the integrity, and in most cases, the confidentiality of the PUF model depending on the security properties required of the enrollment protocol. Consequently, the AS can be classified into three categories based on these security guarantees, as shown in Figure 11. The classification of the AS operational mode is described as follows:

- **Public database:** The authentication server has to guarantee the integrity of the PUF model that can be accessed publicly by any participant.
- **Private database:** The authentication server has to guarantee the integrity and the confidentiality of the PUF model that can only be accessed by the authorized users.
- **Root authenticator:** The authentication server stores the PUF model under one of the previous database modes. Furthermore, it fully plays the role of the verifier as introduced in the 3CE architecture.

#### 3.2.4. Manufacturer

The manufacturer plays the initial role of constructing the prover hardware. He extracts enough challenge–response pairs to construct the PUF model and he sends it securely to the authentication server. These actions mark the end of participation of the manufacturer in the enrollment process.

### 3.3. Threat Models

The adopted threat models in the existing ML model-based PUF authentication protocols can be categorized depending on the accessibility properties of the ML model in question. As described in Section 3.2.3, the *private database* and *root authenticator* modes require the authentication server to keep the PUF model a secret and to only provide access to the trusted users. Thus, the adversary cannot get hold of the PUF model, and he can only attack the system through external actions such as eavesdropping or replaying the messages exchanged between the enrollment entities. This attacker falls into the **outsider threat** category. However, The *public database* mode assumes that any user can obtain the model without any restrictions. The security of this mode is assured by relying on additional assumptions about the attacker’s capabilities. Regarding the adversary’s powers over the communication channel, he is able to eavesdrop on the exchanges between the prover and the verifier or the authentication server depending on the adopted enrollment architecture. Furthermore, he can actively query the PUF holder by its own challenges. This action aims at collecting enough CRPs for the attempted model reconstruction attack in the case of the private operational modes. However, the adversary is assumed unable to conduct invasive attacks on the prover software, which guarantees the correctness of the enrollment protocol execution. This assumption can be assured through the use of lightweight integrity verification of IoT systems such as the remote attestation schemes [52,53]. As a consequence, the adopted threat models are classified as follows:

- **Public model adversary (Pub-Adv)**: The goal of the adversary shifts from modeling the PUF hardware to attacking the additional security mechanisms in order to bypass the authentication process. For example, he can focus on reducing the response generation time using the public PUF model to bypass the time-bound assumption.
- **Private model adversary (Priv-Adv)**: The adversary aims at creating a precise PUF model based on the obfuscated challenge–response exchanges. This ML model serves as a tool to predict the correct responses to the challenges of the verifier as a way to enroll malicious devices.

The two previously detailed attacker categories can be further extended to assess the robustness of the enrollment protocol against an adversary that can get hold of the PUF model that is used in the authentication process. This scenario is considered as an **insider threat** within the information system of a particular organization. The attack is based on an individual with sufficient access privileges who violates the non-disclosure policies by leaking sensitive information, such as the PUF models. These leaks should be impossible to be traced back to this particular individual. This scenario is only applicable in the context of the 4CE architecture where the verifier might be the source of the leakage, since it represents the role with the least level of trust in comparison with the authentication server. On the other hand, the verifier is assumed to be able to properly perform the authentication process without the risk of fraudulently enrolling malicious devices. This is due to the fact that the enrollment process of a particular device can lead back to the individual responsible once the malicious object is discovered. However, the PUF model, is shared between all the potential operators, which eliminates any possibility of discovering the leakage source.

## 4. Enrollment Protocols Analysis

In this section, we study a selection of model-based PUF enrollment protocols based on the previously identified architectures. The different modules that are applied in the components of these schemes are described and detailed. Afterwards, we provide a security overview of the identified weaknesses in the protocol design and we suggest the adequate mitigation.

### 4.1. Time-Bounded Authentication Protocol

**Figure 12:**
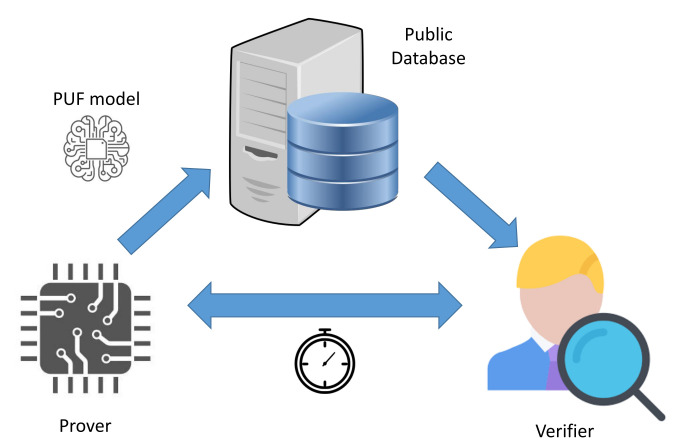
High-Level representation of the time-bound authentication protocol.

This enrollment scheme was proposed in the work of Majzoobi and Koushanfar [54,55] to target the issue of having a public model architecture of the PUF. The security of the protocol is based on the assumption that the time required to generate the responses by PUF hardware is significantly smaller than the time required to predict them using a machine learning model. Thus, it is possible to verify the origin of the received responses by the verifier to avoid any possible ML-based impersonation attacks. The main steps of the time-bound authentication process are illustrated in Figure 12. This proposal is based on the 4CE architecture and adopts the public adversary threat model, which are described, respectively, in Section 3.1 and Section 3.3.

#### 4.1.1. Protocol Components

**Prover**

In this protocol, the operational characteristics of the prover are systematically described based on the following three sub-components:

- **Challenge preparation:** Direct reception.
- **Challenge verification:** The received challenges are not verified.
- **Controlled PUF:**
  i. *PUF specifications:*
    − Nature: Electronic.
    − Architecture: C-RPUF (Section 2.1.3).
  ii. *Reconfigurability:* This option is fully supported.
  iii. *Obfuscation technique:* This mechanism is not applied. The responses are returned to the verifier without any modification.

**Verifier**

The operational characteristics of the verifier are systematically described based on the following two sub-components:

- **Response re-computation:** The verifier applies the public model that is stored in the authentication server, and the desired reconfiguration to predict the responses of the prover.
- **Response verification:** The verifier evaluates the execution time of the challenge–response process. The verification of the PUF output happens only if the responses are received within a pre-fixed time threshold. When the time-bound assumption is satisfied, the response verification process is conducted by a simple bitwise comparison.

**Authentication Server**

The AS in the protocol plays the role of a *public database*. Therefore, the PUF model is also accessible to the adversary. However, the integrity of the stored PUF model is assumed guaranteed.

#### 4.1.2. Security Assessment

**Authentication Property**. *The verifier authenticates the prover only if the time the prover takes to generate the correct response is less than the time-bound threshold.*

To handle the public accessibility to the PUF model, the work of Majzoobi and Koushanfar [55] uses a time-bounded method that prevents the prover from applying a PUF model, since it takes more time than just feeding the challenge as an input to the PUF hardware. In addition, the messages containing the configuration bitstream provide insights about the placements of the specific PUF cells to be used in the case of a reconfigurable PUF. However, the adversary is assumed to be unable to reverse-engineer this information, which prevents him from knowing the used PUF configuration. This assumption suggests that the attacker does not have perfect knowledge of the protocol structure, which partially supports the *Security Through Obscurity* (STO) policy. Thus, this mechanism might be vulnerable to an attack on the distance-bounding protocols [56,57]. Therefore, there is a need for a new method to guarantee that the source of the response is indeed the PUF hardware and not the model used. Since the manufacturer constructs the model of the PUF and stores it in a publicly accessed database, the adversary is assumed to be able to obtain it, just as any legitimate user can. In order to prevent the attacker from using the PUF model to respond to the challenge, the verifier applies a time-bound authentication proof to the challenge–response process based on the assumption that the time required for the response simulation is longer than the time required by the hardware PUF. This assumption is only valid if the minimum response simulation time, represented as $t_{min}^{sim}$, is larger than the upper bound delay for generating an authentic response by the hardware that is represented as $\Delta_{max}$.

The time-bound assumption is based on the computational capabilities of the adversary and the variation in the channel latency to guarantee the correctness of the authentication process. This explains the use of the additional STO assumption about the infeasibility of the attacker decoding the configuration bit-stream, which prevents him from efficiently simulating the behavior of the PUF. In addition, this particular protocol is mainly designed for FPGAs, which makes it unsuitable for the application-specific integrated circuits, such as the majority of the IoT devices. Thus, the reconfigurability technique cannot be applied to thwart the risks of bypassing time-bound authentication.

### 4.2. Slender PUF Protocol

The Slender PUF protocol was proposed in two versions. The conference version was first introduced in the work of Majzoobi et al. [15] to present a new response hiding technique that is based on pattern matching. However, the journal version [46] represents an extension of the response obfuscation through a pseudo-random padding of the selected sub-string. These two proposals are based on the 3CE architecture, and they adopt the private model adversary. The details of these two terms are described, respectively, in Section 3.1 and Section 3.3.

#### 4.2.1. Protocol Components

**Prover**

In this protocol, the operational characteristics of the prover are systematically described based on the following three sub-components:

- **Challenge preparation:** Mutual construction through a nonce exchange.
- **Challenge verification:** The received challenges are not verified.
- **Controlled PUF:**
  i. *PUF specifications:*
    − Nature: Electronic.
    − Architecture: 4-XOR Arbiter PUF (Section 2.1.2).
  ii. *Reconfigurability:* This option is not supported.
  iii. *Obfuscation technique:*
    − **Conference version [15]:** The prover generates a random index ind∈[0,l−1] that represents the first bit of the truncation. Afterwards, he extracts the $l_{sub}$ bits sequence from the *l* bit PUF response to the sent challenges. Then, he sends it to the verifier to validate the enrollment procedure.
    − **Journal version [46]:** The prover conducts the same operations to find the substring response as in the conference version. Then, he generates an additional random $\left(l-l_{sub}\right)$ bit sequence that serves as padding for the substring. Finally, he inserts the truncated response at a random index $ind_{2}\in \left[0,l-l_{sub}-1\right]$ of the generated circular padding sequence.

**Verifier**

The operational characteristics of the verifier are systematically described based on the following two sub-components:

- **Response re-computation:** The verifier uses the PUF secret model that is stored in the authentication server, to precisely compute the expected hardware response.
- **Response verification:** The verification phase is the same for the both versions of the protocol. The verifier tries to find a match between the substring and the simulated PUF response through a maximum sequence alignment. The enrollment is validated under two conditions: the substring alignment should produce a match and the hamming distance between the two sequences should be less than a pre-defined threshold. The latter condition is applied to support the noisiness in the PUF responses.

**Authentication Server**

The AS in the protocol plays the role of a *root authenticator*. Therefore, the PUF model is not accessible to the adversary. Consequently, the authentication server has to guarantee the confidentiality and the integrity of the PUF model.

#### 4.2.2. Security Assessment

**Authentication Property**. *The authentication is successful if the prover response substring matches at some location in the authentication server’s estimated response string within a predefined threshold of time.*

The two versions of the Slender PUF protocol were put to the test in the work of Becker [45]. In this experiment, the author applied the CMA-ES [44] machine learning algorithm, detailed in Section 2.2.4. In the case of the attack on the Slender PUF, Becker targeted the main security assumption of the protocol that the adversary can only compromise the protocol by guessing the truncation indexes, $ind_{1}$ and $ind_{2}$. This assumption aims to establish that the only possible way to model the PUF hardware is to map the substring response sequence to the corresponding challenges. The proposed attack counters this assumption by using a Pearson correlation coefficient corr(.)[58] as a fitness test between the Hamming weights of the generated responses from the parent PUF instances, $HW(R_{i})$, and the Hamming weights of the collected substrings, $HW(W_{i})$. The choice of this fitness function was motivated by the assumption of the higher the computed correlation, the more accurate the PUF instance. This technique efficiently modeled the protected hardware PUF using different levels of noise and two constructions of PUFs (3-XOR and 4-XOR Arbiter PUF). The added noise was applied to simulate the unreliability percentages of the collected hardware PUF responses. The accuracy of the modeled 4-XOR Arbiter PUF reached 97.2% using 600,000 noiseless CRPs. However, the additional 29% noisy responses reduced the accuracy to 92.5% using 1,200,000 samples.

### 4.3. Noise Bifurcation Protocol

The noise bifurcation protocol was introduced in the work of Yu et al. [31] to present a novel response hiding technique. The scheme selects only specific responses to be returned to the verifier. Thus, the attacker is assumed unable to associate the challenges and their corresponding responses. The proposal is based on the 3CE architecture, and it adopts the private model adversary. The details of these two terms are described, respectively, in Section 3.1 and Section 3.3.

#### 4.3.1. Protocol Components

**Prover**

**Figure 13:**
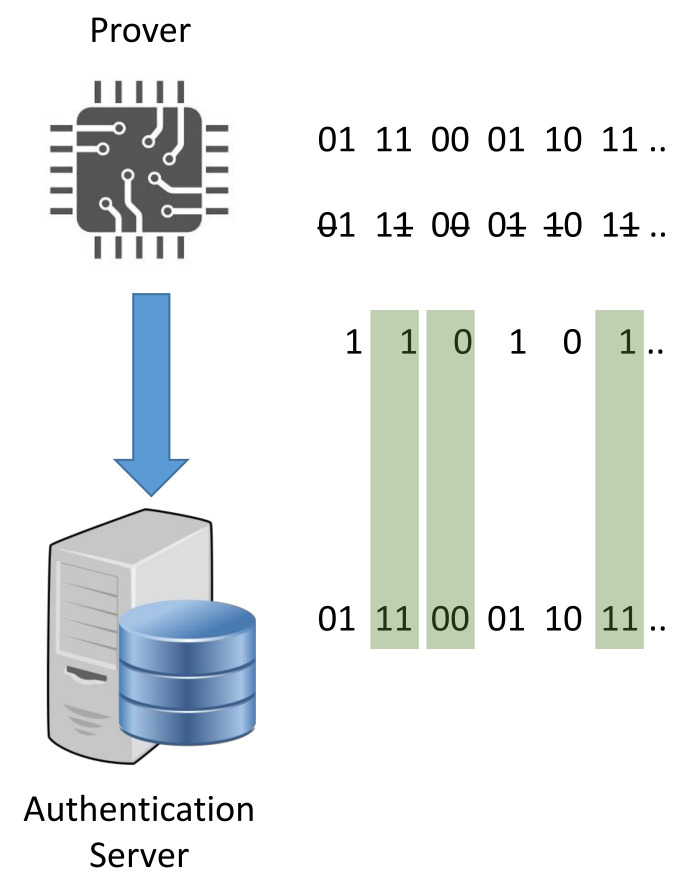
Noise-Bifurcation obfuscation technique.

In this protocol, the operational characteristics of the prover are systematically described based on the following three sub-components:

- **Challenge preparation:** Mutual construction through a challenge exchange. The master challenges are referred to, respectively, as $C_{p}$ for the one generated by the prover and $C_{v}$ for the one generated by the verifier.
- **Challenge verification:** The received challenges are not verified.
- **Controlled PUF:**
  i. *PUF specifications:*
    − Nature: Electronic.
    − Architecture: 4-XOR Arbiter PUF with multiple derivative challenges (Section 2.1.2).
  ii. *Reconfigurability:* This option is not supported.
  iii. *Obfuscation technique:* The prover generates a random challenge $C_{p}$ that represents the second master challenge. Then, he extracts a set of *m* challenges from $C_{p}$ and $C_{v}$. The resulting *m* responses $R\in {\left\{0,1\right\}}^{m}$ is divided into $\frac{m}{d}$ groups of *d* elements (in [31], d=2). Afterwards, only one response per group is randomly chosen and they are returned as a reply to the verifier. The previously described obfuscation technique is illustrated in Figure 13.

**Verifier**

The operational characteristics of the verifier are systematically described based on the following two sub-components:

- **Response re-computation:** The verifier uses the PUF secret model to precisely compute the expected hardware response.
- **Response verification:** The verifier reconstructs the $\frac{m}{d}$ groups using the recomputed responses. Then, he selects the matching responses with the same group and performs the comparison with received results, as highlighted in green in Figure 13. The authentication is successful only when the hamming distance between the selected and the received responses is below a pre-defined tolerance threshold.

**Authentication Server**

The AS in the protocol plays the role of a *root authenticator*. Therefore, the PUF model is not accessible to the adversary. Consequently, the authentication server has to guarantee the confidentiality and the integrity of the PUF model. Most importantly, the delivery of the secret model should be only allowed for the authorized users. Additionally, the AS plays the role of the verifier in the enrollment process.

#### 4.3.2. Security Assessment

**Authentication Property**. *The prover is authentic if the number of mismatched bits, that are computed by the authentication server, are lower than a pre-defined threshold.*

The noise bifurcation protocol have been assessed in the work of Tobisch and Becker [59] through the re-execution of the evaluation methodologies presented in the original paper [31]. The modeling attack focuses on the *full-response replication* strategy to construct the CRP dataset. This technique aims at associating each bit response with the *d* challenges of the corresponding group. However, this assessment revealed some contradictions with the original results published in [31] that is due to the lack of specifications about the applied PUF construction. The original work exploited a XOR Arbiter PUF where each XOR stage receives a random unique challenge. This specific architecture is considered as an additional countermeasure that has not been clearly described. The evaluation of the noise bifurcation technique on a classical PUF construction, where the same challenge is applied to all the stages, reveled that the obfuscation scheme does not prevents the adversary from modeling the PUF. The attack was conducted using the logistic regression model with a considerable number of CRPs that depends on the number of XOR stages with an accuracy that varies between 84% and 92%. The details of the applied ML technique are described in Section 2.2.1.

### 4.4. OB-PUF Protocol

The OB-PUF protocol was introduced in the work of Gao et al. [60] to present a challenge obfuscation technique. The main objective behind the scheme is to prevent the adversary from constructing a sound CRP dataset that is, eventually, used to model the PUF behavior. On the other hand, the legitimate verifier holds the PUF model that is used to authenticate the prover based on the received responses. The proposal is based on the 3CE architecture and it adopts the private model adversary. The details of these two terms are described, respectively, in Section 3.1 and Section 3.3.

#### 4.4.1. Protocol Components

**Prover**

**Figure 14:**
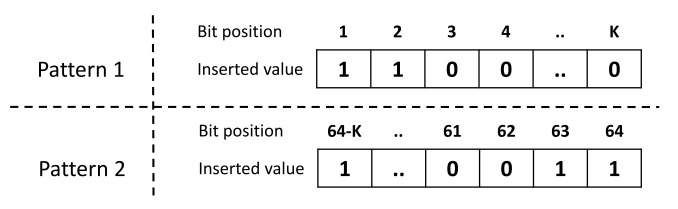
Two pattern examples that might be added to the obfuscated challenge.

In this protocol, the operational characteristics of the prover are systematically described based on the following three sub-components:

- **Challenge preparation:** Direct reception.
- **Challenge verification:** The received challenges are not verified.
- **Controlled PUF:**
  i. *PUF specifications:*
    − Nature: Electronic.
    − Architecture: Arbiter PUF (Section 2.1.1).
  ii. *Reconfigurability:* This option is not supported.
  iii. *Obfuscation technique:* The prover receives the obfuscated challenge $C_{OB}\in {\left\{0,1\right\}}^{l-k}$ that is sent by the verifier where *l* is the challenge bit-length (e.g., l=64) and *k* is the number of the obfuscated bits. Afterwards, he randomly chooses the pattern of the additional *k* bits and executes them using the PUF hardware to obtain a *n*-bit response *R* where *n* is the number of Arbiter PUF instances onboard the prover. The pattern is a set of *k* pre-defined bit values and indices that are used as a padding to the obfuscated challenge, as highlighted in Figure 14. The response *R* is, then, returned to the verifier.

**Verifier**

The operational characteristics of the verifier are systematically described based on the following two sub-components:

- **Response re-computation:** The verifier uses the PUF secret model to compute all the possible responses of the obfuscated challenge based on all the pre-defined padding patterns.
- **Response verification:** The verifier compares the received response with all the predicted responses to authenticate the prover.

**Authentication Server**

The AS in the protocol plays the role of a *root authenticator*. Therefore, the PUF model is not accessible to the adversary. Consequently, the authentication server has to guarantee the confidentiality and the integrity of the PUF model. Most importantly, the delivery of the secret model should be only allowed for the authorized users. Additionally, the AS plays the role of the verifier in the enrollment process.

#### 4.4.2. Security Assessment

**Authentication Property**. *The authenticity of the prover is established if the candidate emulated response for the given obfuscated challenge $C_{OB}$ is the same as the received response R.*

The security of the OB-PUF protocol was compromised in the work of Delvaux [61]. The attack strategy is based on the direct interaction with the prover that is holding the PUF. The main objective of the adversary is to search for the obfuscated challenges $C_{OB}$ that generate similar results. This process is conducted through the repetitive execution of the same obfuscated challenges for a specific number of times and the assessment of the resulting responses. The collected CRPs serve as a dataset to construct the ML model of the PUF using logistic regression. The details of the applied ML technique are described in Section 2.2.1.

The original work [60] claimed that the adversary cannot exceed the accuracy limit of 72% even after collecting $10^{6}$ random CRPs which is not sufficient to bypass the authentication. However, the described strategy provided the attacker with the ability to reach an 85% accuracy using the same ML technique. Afterwards, the attacker extended their strategy to use the constructed model to build a new dataset using uniformly distributed challenges. This procedure increased the accuracy of the adversarial model to reach 95%. This attack could have been mitigated through the application of a challenge verification procedure on the prover side that eliminates the repetitive execution of the same obfuscated challenge. This could be done by the use of an approximate set membership test such as the XOR filter [62].

### 4.5. Lightweight PUF-Based Authentication Protocol

The lightweight PUF authentication protocol was introduced in the work of Yilmaz et al. [63] to present a suitable enrollment protocol for the resource-constrained devices. The main objective behind the scheme is to reduce the power and memory consumption with respect to the legacy IoT protocol DTLS handshake authentication. The proposal is based on the 4CE architecture and it adopts the private model adversary. The details of these two terms are described, respectively, in Section 3.1 and in Section 3.3.

#### 4.5.1. Protocol Components

**Prover**

In this protocol, the operational characteristics of the prover are systematically described based on the following three sub-components:

- **Challenge preparation:** Direct reception.
- **Challenge verification:** The received challenges are not verified.
- **Controlled PUF:**
  i. *PUF specifications:*
    − Nature: Electronic.
    − Architecture: Arbiter PUF (Section 2.1.1).
  ii. *Reconfigurability:* This option is not supported.
  iii. *Obfuscation technique:* The prover uses the RC5 encryption scheme [64] to encrypt the MAC address of the device with the response of the PUF *R*. The returned value of the prover is formulated as RC5(MAC,R⊕T) where the *T* parameter is the timestamp which guarantee the freshness of the obfuscation procedure.

**Verifier**

- **Response re-computation:** The verifier uses the PUF secret model to precisely compute the expected hardware response.
- **Response verification:** The verifier predicts the PUF response through the use of the secret model. Then, he computes the expected output value using the predicted PUF response and the timestamp. Afterwards, he compares the two ciphertexts to validate the authentication process.

**Authentication Server**

The AS in the protocol plays the role of a *private database*. Therefore, the PUF model is not accessible to the adversary. Consequently, the authentication server has to guarantee the confidentiality and the integrity of the PUF model. Most importantly, the delivery of the secret model should be only allowed for the authorized users.

#### 4.5.2. Security Assessment

**Authentication Property**. *The prover is authenticated if the verifier validates the received RC5 ciphertext using the PUF model response and the timestamp.*

The obfuscation technique is based on the RC5 encryption scheme. The security of the procedure is based on the infeasibility to access the PUF responses by an adversary that does not have the accurate model. However, this encryption scheme has requirements regarding the length of the applied key (suggested 128 bits) which is not clearly the case in the original protocol simulation. One study [63] implemented the authentication scheme using a PUF architecture that provide response bit-lengths that vary, respectively, between 16 and 32 bits. Thus, the confidentiality of the sent ciphertext might be compromised through the correlation attack [65] or the timing attack [66]. In addition, the use of an encryption scheme to obfuscate the PUF response without error-correcting codes affects drastically the usability of the protocol. This is due to the non-ideal reliability of the PUF hardware that might produce bit-flips in the responses. Consequently, these incidents result in errors in the decryption process on the verifier side. Furthermore, the PUF model predictions might not be always 100% accurate which ruins the de-obfuscation process.

### 4.6. RF-PUF Protocol

The RF-PUF protocol was introduced in the work of Chatterjee et al. [26] to present an ANN-based process to authenticate the wireless nodes. The details of the applied ML technique are described in Section 2.2.3.

**Figure 15:**
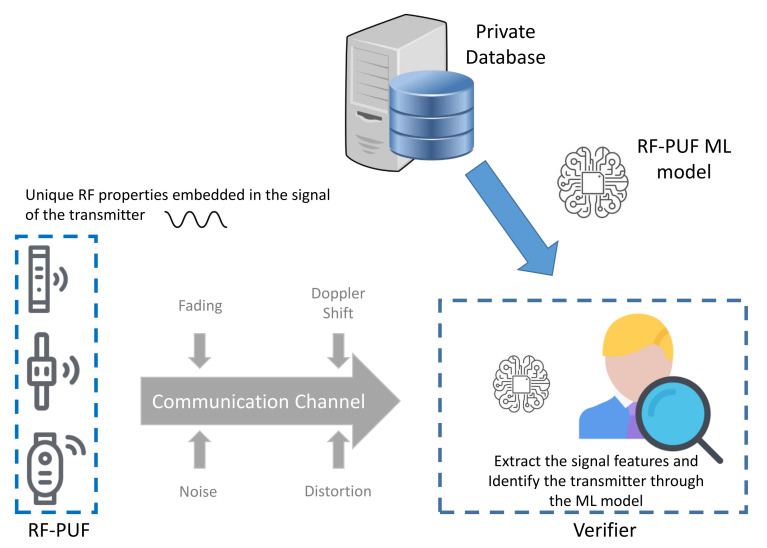
High-Level representation of the RF-PUF protocol.

Similar to the concept of the hardware PUFs, the proposal uses the effects of inherent variation on radio-frequency properties of the wireless transmitters Tx (provers). The detection is based on a machine learning model at the receiver side Rx (verifier). The main objective behind the scheme is to distinguish between the signals received by the provers in order to uniquely identify them, as illustrated in Figure 15. The proposal is based on the 4CE architecture and it adopts the private model adversary. The details of these two terms are described, respectively, in Section 3.1 and Section 3.3.

#### 4.6.1. Protocol Components

**Prover**

In this protocol, the operational characteristics of the prover are systematically described based on the following three sub-components:

- **Challenge preparation:** Direct reception.
- **Challenge verification:** The received challenges are not verified.
- **Controlled PUF:**
  i. *PUF specifications:*
    − Nature: Non-electronic.
    − Architecture: RF-PUF.
  ii. *Reconfigurability:* This option is not supported.
  iii. *Obfuscation technique:* This mechanism is not applied. The responses are returned to the verifier without any modification.

**Verifier**

- **Response re-computation:** This option is not supported.
- **Response verification:** The verifier identifies the transmitters through their radio signatures and the ANN model.

**Authentication Server**

The AS in the protocol plays the role of a *private database*. Therefore, the PUF model is not accessible to the adversary. Consequently, the authentication server has to guarantee the confidentiality and the integrity of the PUF model. Most importantly, the delivery of the secret model should be only allowed for the authorized users.

#### 4.6.2. Security Assessment

**Authentication Property**. *The prover is authenticated if the verifier validates the RF signature of the prover through the ANN model.*

The RF-PUF is based on a machine learning model that identifies specific communication nodes through a set of propagation properties (local oscillator frequency, channel information, DC offset and I-Q mismatch on the transmitter side). The model is trained using a dataset of challenge–response pairs that are collected from a group of different transmitters. The challenge is a pre-defined bit-sequence that is transmitted to the receiver node. The corresponding response is represented as a set of propagation features that are extracted from the challenge transmission. The model is trained to distinguish between a number of transmitters with a high accuracy under varying channel conditions. The RF-PUF protocol can authenticate up to 10,000 devices with an accuracy of 99%. However, the decommissioning of the deployed devices poses a serious threat to the security of the protocol. This is explained by the unfeasability of removing a specific device from the list of accepted identities. This operation can be conducted by retraining the model from scratch without using the CRP dataset of that decommissioned device which is computationally costly, especially when managing a big number of IoT objects.

The authors in [26] have discussed the possibility of facing an attacker that tries to mimick a specific transmitter through the use of a machine learning model. The adversarial model in question intends to produce the same transmission signature as the target transmitter through the collection of a sufficient number of CRPs. The paper argues that the adversary cannot associate the collected CRPs to their corresponding identities when he eavesdrops on a multi-device environment. Therefore, the attacker requires a larger dataset to enhance the accuracy of their unsupervised learning model. However, the unidentified CRPs can be indexed when we take under consideration the insider threat scenario where an adversary can obtain the ANN identification model. Thus, it transforms back the problem into a supervised learning procedure that facilitates the mimicking attack.

### 4.7. Set-Based Obfuscation Protocol

The Set-Based Obfuscation protocol was introduced in the work of Zhang and Shen [67] to present an obfuscation technique that resists the existing ML modeling attacks. The introduced methodology relies on the use of a secret set of CRPs that is stored on the authentication server and on the prover. These obfuscation CRPs serve as a way to modifiy the inputs and outputs of the PUF to reinforce the complexity of the PUF mapping function. The proposal is based on the 3CE architecture and it adopts the private model adversary. The details of these two terms are described, respectively, in Section 3.1 and Section 3.3.

#### 4.7.1. Protocol Components

**Prover**

In this protocol, the operational characteristics of the prover are systematically described based on the following three sub-components:

- **Challenge preparation:** Direct reception.
- **Challenge verification:** The received challenges are not verified.
- **Controlled PUF:**
  i. *PUF specifications:*
    − Nature: Electronic.
    − Architecture: Arbiter PUF Section 2.1.1.
  ii. *Reconfigurability:* This option is not supported.
  iii. *Obfuscation technique:* Random set-based obfuscation (RSO). The obfuscation challenges are stored in the Non-Volatile Memory (NVM). The prover selects randomly two challenges from a set *K* to be applied to the PUF in order to generate the obfuscation keys, $Key_{i}$ and $Key_{j}$. Afterwards, the received challenges are XORed with $Key_{i}$ to modify the input $C^{\prime }$. Furthermore, the output $R^{\prime }$ is XORed with $Key_{j}$. The computed response $\hat{R}$ is split into two $\frac{n}{2}$-bit responses $\left(\hat{R_{a}},\hat{R_{b}}\right)$ where *n* is the bitlength of $\hat{R}$. Finally, the $\hat{R_{b}}$ response is transmitted to the verifier.

**Verifier**

- **Response re-computation:** The verifier uses the PUF secret model and the set of obfuscation CRPs to compute all the potential responses.
- **Response verification:** The verifier compares the received response to the computed set of potential responses. The enrollment is successful if the verifier finds two responses where the number of mismatched bits is less than a pre-defined threshold.

**Authentication Server**

The AS in the protocol plays the role of a *root authenticator*. Therefore, the PUF model is not accessible to the adversary. Consequently, the authentication server has to guarantee the confidentiality and the integrity of the PUF model. Most importantly, the delivery of the secret model should be only allowed for the authorized users. Additionally, the AS plays the role of the verifier in the enrollment process.

#### 4.7.2. Security Assessment

**Authentication Property**. *The prover is authenticated if the authentication server finds a candidate simulated response that has a bit-rate mismatch with the received prover response which is lower than a pre-defined threshold.*

The RSO obfuscation technique has been proven resilient against the existing ML modeling attacks, such as LR, SVM, ANN and CMA-ES. The modeling accuracy has been reduced to a limit closer to 50%, which is equivalent to a random guess. This technique requires the storage of the obfuscation CRPs on both the AS and the prover. Each obfuscation challenge consists of a list of *n* sub-challenges. Therefore, the total number of used sub-challenges is m×n. Thus, the storage space is estimated to be m×n×n bits. In order to achieve the maximum level of security that the protocol can offer, the recommended number of bits according to the original paper [67] is n=128. Thus, the required storage space is directly dependent on the number of the obfuscation challenges *m* that is controlled by the user. For example, in the case m=1000, the required NVM memory space is 16 Megabits, which is not suitable for resource-constrained devices. On the other hand, the use of less obfuscation challenges may affect the performance of the RSO scheme against the modeling attacks. This is explained by the application of the set-updating mechanism [67], which updates the set of obfuscation challenges located in set *K*. Therefore, there is a need to study the effect of the repetitive use of obfuscation challenges.

## 5. Discussion and Future Research Directions

In this section, we discuss the highlighted results in Table 1. The state-of-the-art model-based PUF protocols have adopted one of the two architectures presented in Section 3.1. The 3CE architecture, which is used by a number of protocols in Section 4, requires the IoT object (prover) to communicate directly with the remote authentication server. Thus, the obligation to connect the device to the network prior to the authentication procedure presents a potential threat. In addition, this centralized architecture relies on the AS as the root authenticator. Therefore, it increases the workload for the server and limits the scalability in comparison with the decentralized version that is the 4CE architecture. However, the delivery of the PUF model to the verifier nodes to perform the authentication can result in the leakage of this secret.

This insider threat is justified by the risk of delegating the sensitive enrollment information to a trusted device with a lower level of security compared to the AS. The existing 4CE enrollment protocols described in Section 4 have not taken in consideration this insider threat model. However, the time-bound authentication protocol, as described in Section 4.1, has demonstrated constrained resistance based on the reconfigurability parameter, the attacker computational power and the characteristics of the used communication channel. In addition, we have studied the discovered vulnerabilities in the existing model-based PUF enrollment protocols that affect the ousider threat resistance. As detailed in Section 4, a number of these attacks are the results of weakness in constructing the response obfuscation technique or the use of a vulnerable cryptographic scheme. However, some other vulnerabilities are the consequences of the lack of a challenge verification mechanism that would verify the validity of the received challenges by the prover, as in the case of the OB-PUF protocol.

The design process of the model-based PUF enrollment protocol can be enhanced through the use of our proposed architectures and the attacker models. The building block diagrams in the 3CE and 4CE structure can help future researchers to design and assess the components of the protocols independently. Furthermore, they facilitate creating a mitigation procedure related to an attack on a specific component of the authentication process. We have shown an example of an attack on the obfuscation technique of the OB-PUF protocol that could have been mitigated through the implementation of a challenge verification component. Unfortunately, in our study we noticed that this component is generally overlooked by protocol designers, as shown in Table 1. Moreover, the insider threat resistance is still an open research question, since it cannot be fully guaranteed by the existing model-based PUF enrollment protocols, as illustrated in Table 1.

In the insider threat scenario, the leaked PUF ML model can be used to successfully bypass the authentication procedure. Therefore, there is a need for an identification mechanism to recognize the use of that specific model during an enrollment session. The use of ML watermarking techniques [69,70] represents a promising solution to performing this particular task. However, all of these existing watermarking methods target mainly the digital media classification models (images, videos or sounds), and they cannot be used for PUF models. This is explained by the nature of the PUF circuit, which takes as an input a random bit sequence challenge. For instance, the application of an out-of-distribution input challenge as a trigger (the trigger is an input sample that is intentionally assigned a wrong label by the watermarked model) cannot be adopted in our case because every combination of bits belongs to the challenge set ${\left\{0,1\right\}}^{l}$. Moreover, any kind of modification to the challenge bit sequence directly modifies the labeled response, and consequently affects the prediction accuracy of the PUF model. This is explained by the difficulty of changing the high likelihood response prediction of a random challenge without reducing the overall performance of the watermarked model. Thus, it is no longer possible to learn the correct behavior of the PUF circuit. Consequently, there a need for a specifically crafted watermarking technique for the case of the binary output PUF models.

## 6. Conclusions

In this survey, we have focused on the usage of ML models of PUF circuits for performing the enrollment process due to their scalability advantages. The use of a mathematically clonable PUF requires the adoption of additional security measures to prevent the modeling attacks through the collection of CRPs. This operation is considered quite complex to address without having a clear idea of the different entities of the protocol and their respective components. Therefore, we have introduced two enrollment architectures that map the different nodes participating in the authentication process of the IoT devices. We have studied a selection of model-based PUF enrollment protocols and we have outlined their security limitations with respect to the identified design flaws.

The proposed architectures facilitate the mitigation of some of the highlighted weakness by modifying vulnerable components in the protocol design. The building block diagrams in the 3CE and 4CE structures can help future researchers to design and independently assess the system components of the protocols. The resiliency of the selected enrollment schemes has been assessed against an insider threat within the organization.

This study yielded that these protocols cannot fully guarantee the security of the enrollment procedure when the PUF model is leaked to the adversary. Thus, there is a need for an identification mechanism to recognize the use of that specific ML model during an enrollment session instead of the legitimate hardware. The use of a distance-bounding protocol with a fixed time-bound has been previously proposed to distinguish between the use of a PUF hardware and an ML model. However, this technique requires the accurate estimation of a dynamic time-bound that is based on the characteristic of the communication channel and the complexity of the used PUF circuit. In addition, the use of a watermarking technique in the PUF model can be a potential solution to the information leakage problem. This attack scenario is generally overlooked by protocol designers, and mitigation of insider threats is still an open discussion.

## Figures and Tables

**Table 1:** Summary of the studied enrollment protocols.

Protocol	Architecture	Prover					Verifier		Authentication Server	Security Assessment	
		*Challenge Preparation*	*Challenge Verification*	*CPUF*			*Response Re-Computation*	*Response Verification*		*Outsider Threat Resistance*	*Insider Threat Resistance*
				PUF Construction	Reconfigurability	Obfuscation Technique					
Time-bounded Authentication Protocol [54,68]	4CE	Direct Reception	n/a	C-RPUF	Yes	n/a	Yes	Time-bound Verification Bitwise Comparison	Public Database	Partially Yes	Partially Yes
Slender PUF Protocol [15,46]	3CE	Mutual Construction	n/a	4-XOR Arbiter PUF	No	Substring Matching	Yes	Response Correlation	Root Authenticator	No	-
Noise Bifurcation Protocol [31]	3CE	Mutual Construction	n/a	4-XOR Arbiter PUF	No	Noise Bifurcation	Yes	Bitwise Comparison	Root Authenticator	No	-
OB-PUF Protocol [60]	3CE	Direct Reception	n/a	Arbiter PUF	No	Obfuscated Challenge Insertion	Yes	Bitwise Comparison	Root Authenticator	No	-
Lightweight PUF-Based Authentication Protocol [63]	4CE	Direct Reception	n/a	Arbiter PUF	No	Encryption	Yes	Ciphertext Comparison	Private Database	Partially Yes	No
RF-PUF Protocol [26]	4CE	Direct Reception	n/a	RF-PUF	No	n/a	No	ANN Model	Private Database	Yes	No
Set-Based Obfuscation Protocol [67]	3CE	Direct Reception	n/a	Arbiter PUF	No	Random Set-based Obfuscation	Yes	Bitwise Comparison	Root Authenticator	Yes	-

## Data Availability

Not applicable.

## Abbreviations

The following abbreviations are used in this manuscript:

ANN	Artificial Neural Network
AS	Authentication Server
CC	Communication Channel
CMA-ES	Covariance Matrix Adaptation-Evolutionary Strategies
CP	Challenge Preparation
CPUF	Controlled PUF
CRP	Challenge–Response Pair
CV	Challenge Verification
DAPUF	Double Arbiter PUF
ES	Evolutionary Strategies
ETSI	European Telecommunications Standards Institute
FPGA	Field-Programmable Gate Array
IoT	Internet of Things
LR	Logistic Regression
LR-PUF	Logically Reconfigurable PUF
ML	Machine Learning
MLP	Multi-Layer Perceptron
NIST	National Institute of Standards and Technology
NVM	Non-Volatile Memory
OTP	One-Time Password
Priv-Adv	Private Model Adversary
PUF	Physical Unclonable Function
Pub-Adv	Public Model Adversary
RoT	Root of Trust
RSO	Random Set-based Obfuscation
SLP	Single Layer Perceptron
STO	Security Through Obscurity
SVM	Support Vector Machine
3CE	Three-Component Enrollment
4CE	Four-Component Enrollment
